# The impact of socioeconomic status on emergency department outcome in a low-income country setting: A registry-based analysis

**DOI:** 10.1371/journal.pone.0223045

**Published:** 2019-10-16

**Authors:** Vijay C. Kannan, Giannie N. Rasamimanana, Victor Novack, Lior Hassan, Teri A. Reynolds

**Affiliations:** 1 Department of Emergency Medicine, Beth Israel Deaconess Medical Center, Boston, MA, United States of America; 2 Emergency and Intensive Care Unit, Centre Hopitalier de Professeur Zagaga, Mahajanga, Madagascar; 3 Clinical Research Center, Soroka University Hospital and Faculty of Health Sciences, Ben-Gurion University of the Negev, Beer Sheba, Israel; 4 Department of Emergency Medicine, University of California, San Francisco, CA, United States of America; Newcastle University, UNITED KINGDOM

## Abstract

**Background:**

The impact of socioeconomic status on health has been established via a broad body of literature, largely from high-income countries. Investigative efforts in low- and middle-income countries have suffered from a lack of reporting standardization required to draw comparisons across countries of varying economic strata. In this study we aimed to evaluate the impact of socioeconomic status on emergency department outcomes in a low-income African country using international data classification systems.

**Methods:**

This was a retrospective cohort study was conducted at a tertiary care center in northern Madagascar. Data were abstracted from paper charts into an electronic registry using Integrated Public Use Microdata Series codes for occupation, Nam-Powers-Boyd (NPB) scores for socioeconomic status, and Clinical Classifications Software ICD-9 equivalents for diagnosis. Outcome was dichotomized to the combined disposition of death or transfer directly to operating theater (OT) versus discharge. We used t-tests to compare baseline characteristics between these groups. We used chi-square analysis to test the association between occupational class and diagnosis. Finally, multivariate logistic regression analysis was performed examining the impact of NPB score on death/OT outcome, adjusting for age, gender, diagnosis and occupation.

**Results:**

5271 patients were seen during the 21-month study period with a death/OT rate of 9.7%. Older age and male gender were more common in death/OT patients (both p<0.001), and were shown to have positive odds ratios for this outcome in multivariate modeling (p<0.006 and <0.001). Occupational class was found to influence diagnosis for all classes (p<0.001) except Sales and Office. Adjusting for these 3 factors, we found a strong independent association between NPB quartile and death/OT outcome. Relative to the 1^st^ quartile, the odds ratio in the 4^th^ quartile was 2.9 (p = 0.004), the 3^rd^ quartile 1.8 (p = 0.094), and the 2^nd^ quartile 3.1 (p<0.001).

**Conclusion:**

To our knowledge, this is the first Malagasy study describing the relationship between socioeconomic status on emergency care outcomes. We found a stronger effect on health in this setting than in high-income countries, highlighting an important healthcare disparity. By using standardized classification systems we hope this study will serve as a model to facilitate future comparative efforts.

## Introduction

Occupation exerts its influence on health via both direct and indirect mechanisms. It directly affects the chance of illness or injury via self-evident risks such as dangerous working conditions or high exertion. Its more subtle indirect effect is through the relationship between occupation and socioeconomic status (SES), and the impact of SES on outcomes once an illness or injury has occurred. SES has been shown to independently affect health via a broad body of literature, independently impacting mortality, morbidity, and length of stay.[1,2] This body of literature largely originates from high-income countries (HICs). The impact of SES on health in low- and middle-income countries (LMICs) is less clear, and may in fact be quite different owing to greater income disparities, lower absolute income, and other factors.[3] Investigative efforts have thus far suffered from a lack of reporting standardization required to draw comparisons across countries of varying economic strata, with some studies using measures such as health insurance status whereas others use average income in a given census tract.[4,5] Yet another barrier is that the lack of data linkage in LMICs makes it difficult to tie SES demographic data with clinical outcome. The Nam-Powers-Boyd (NPB) scale provides a standard to facilitate this comparison.

The NPB scale was developed by the United States Census Bureau in 1960 and is currently on its 7^th^ iteration. It is an occupation-based measure of socioeconomic status that assigns a 0–100 score based occupational class, reflective of average income and required education.[6] It categorizes occupational class using the Integrated Public Use Microdata Series (IPUMS) 2010–2012 American Community Surveys (ACS) taxonomy. This is a multi-level system that groups individual occupations (e.g. miner, nurse) into larger occupational classes (e.g. Extraction Workers, and Healthcare Practitioners, respectively). This structure confers two advantages when examining the impact of SES on health. First, it allows researchers to adjust for the direct effects of occupation on health (e.g. occupational hazards) in order to isolate the effect of SES amongst equal scoring-professions across classes. Second, it facilitates future international comparisons by using the IPUMS taxonomy, which has equivalent occupational codes for 82 different countries.

This study aims to assess the impact of NPB-defined SES on clinical outcome via multivariate regression analysis adjusting for age, sex, diagnosis, and occupational class for patients presenting to a combined emergency department and intensive care unit in northern Madagascar. The outcome of interest is the combined endpoint of mortality or procession directly to the operating theater, which is used as a marker of acuity. Admission status cannot be used as a measure of acuity because patients in this setting are often admitted to have a diagnostic test performed, rather than as the result of the findings from a diagnostic test–a key difference in acuity indicators between low and high income settings. The World Bank ranks Madagascar as the 6^th^ poorest country in the world by gross domestic product (GDP).[7] Its wealth is the 55^th^ most unequally distributed overall by the Gini index.[8] There are currently no reports on the impact of SES on the health of its inhabitants.

## Materials and methods

This was a retrospective cohort study was performed in the combined Emergency and Intensive Care Unit (SUSI) at the University Hospital of Mahajanga (CHU P-ZAGA), a 400-bed hospital serving the Boeny region with a catchment population of an estimated 543,000 persons. This was a retrospective cohort study of all patients presenting to the SUSI between 1 January 2011 and 30 September 2012. The SUSI does not see pediatric patients. Data were recorded in a paper registry booklet by attending and resident physicians. These data were later entered into the electronic SUSI registry by the charge nurse, even if the patient was dead on arrival and received no care. If data were missing or illegible, an attempt was made to contact the treating physician to rectify this, but data points for which this was unsuccessful were omitted from electronic entry. Data were transcribed directly into the registry from the registration booklet, there was no intermediary data abstraction form. Transcription was done on a weekly basis. The charge nurse was trained in how to do this data entry by the chief of service directly. Data collected included age, sex, profession, diagnosis, and disposition. Profession was self-reported by the patient or, in cases in which a patient was unconscious or altered, by their next of kin. As this was a retrospective review of de-identified registry data, the need for patient consent was waived by the Institutional Review Committee of Research at CHU P-ZAGA, who approved this study.

Profession was manually converted to corresponding Integrated Public Use Microdata Series (IPUMS) 2010–2012 American Community Survey occupational classification system codes (ACS OCC Codes). The corresponding 0–100 score on the Nam-Powers-Boyd (NPB) Occupational scale was subsequently assigned.

Diagnosis was manually converted into corresponding Clinical Classifications Software multi-level categories, which have direct International Classification of Diseases (ICD) equivalents. Aggregate categories of disease were compiled according to the WHO Global Health Estimates top 5 causes of mortality in Madagascar–Trauma, Infectious Disease, Mental Health, Neoplasm, and Noncommunicable Disease (see Supporting Information S1 Table).

Patients at this site may be discharged, die, go directly to the operating theater, be admitted to wards, or be admitted to the ICU which is an unit within the ED itself and not clearly differentiated in reporting. Accordingly, we are unable to separate out ED vs. ICU patients out as a marker of acuity. Therefore we erred towards the null hypothesis by selecting an extreme marker of acuity by creating a combined endpoint of death or proceeding directly to operating theater as a severe outcome. Unlike in HICs, hospital admission is not a marker of acuity in the LMIC setting. This is due to the fact that patients are often admitted to receive a certain laboratory or imaging test, rather than due to clinical acuity. Accordingly, admitted patients were not included in our outcome comparison (death/OT vs. discharge) but were included in the “All” category.

Data are either expressed as median ± interquartile range (IQR) or by number and percentage. To compare patient characteristics by disposition we used an independent t-test with an assumed normal distribution or a Mann-Whitney test if parametric assumptions could not be satisfied. Chi-square tests were used to compare categorical variables such as occupation and diagnosis. We conducted two logistic regression models: 1) we examined the association of one’s NPB score on the combined endpoint of death or transfer directly to operating theater, 2) we performed the same analysis on our large student population, which does not have a corresponding ACS code or NPB score. We included all clinically and statistically significant variables into the models–the inclusion criterion was a p-value <0.20. Statistical analyses were performed using the SPSS package 23^rd^ edition. (IBM/SPSS, Chicago, IL). P-values of <0.05 (two–sided) were considered to be significant.

## Results

Table 1 reports patient characteristics by outcome. There were 5,271 patients seen during the study period. Median age was 31 years (IQR 21–47) and 54% were male. The median Nam-Powers-Boyd score was 28 (IQR 15–42). Trauma was the overall most common aggregate disease category, followed by noncommunicable disease and infectious disease. The highest rate of death/OT occurred in patients with Noncommunicable disease, followed by infectious disease and then trauma.

**Table 1:** Demographic characteristics.

	Death/OT n = 509	Discharge n = 2185	All n = 5271	P-value
**Median Age** **yrs (IQR)**	42(59–24)	25(36–18)	31(47–21)	<0.001
**Male Sex** **n (%)**	316(62.1)	1134(51.9)	2827(53.7)	<0.001
**Median NPB** **n (IQR)**	32(42–20)	23(42–15)	28(42–15)	<0.001
**Aggregate Diagnoses** **n (column %)**				
**Trauma**	101(19.8)	1472(67.4)	2273(44.2)	
**Infectious Disease**	132(25.9)	113(5.2)	754(14.7)	
**Mental Health**	19(3.7)	349(16)	506(9.8)	
**Neoplasm**	12(2.4)	5(0.2)	63(1.2)	
**Noncommunicable disease**	245(48.1)	234(10.7)	1542(30)	

**Fig 1:**
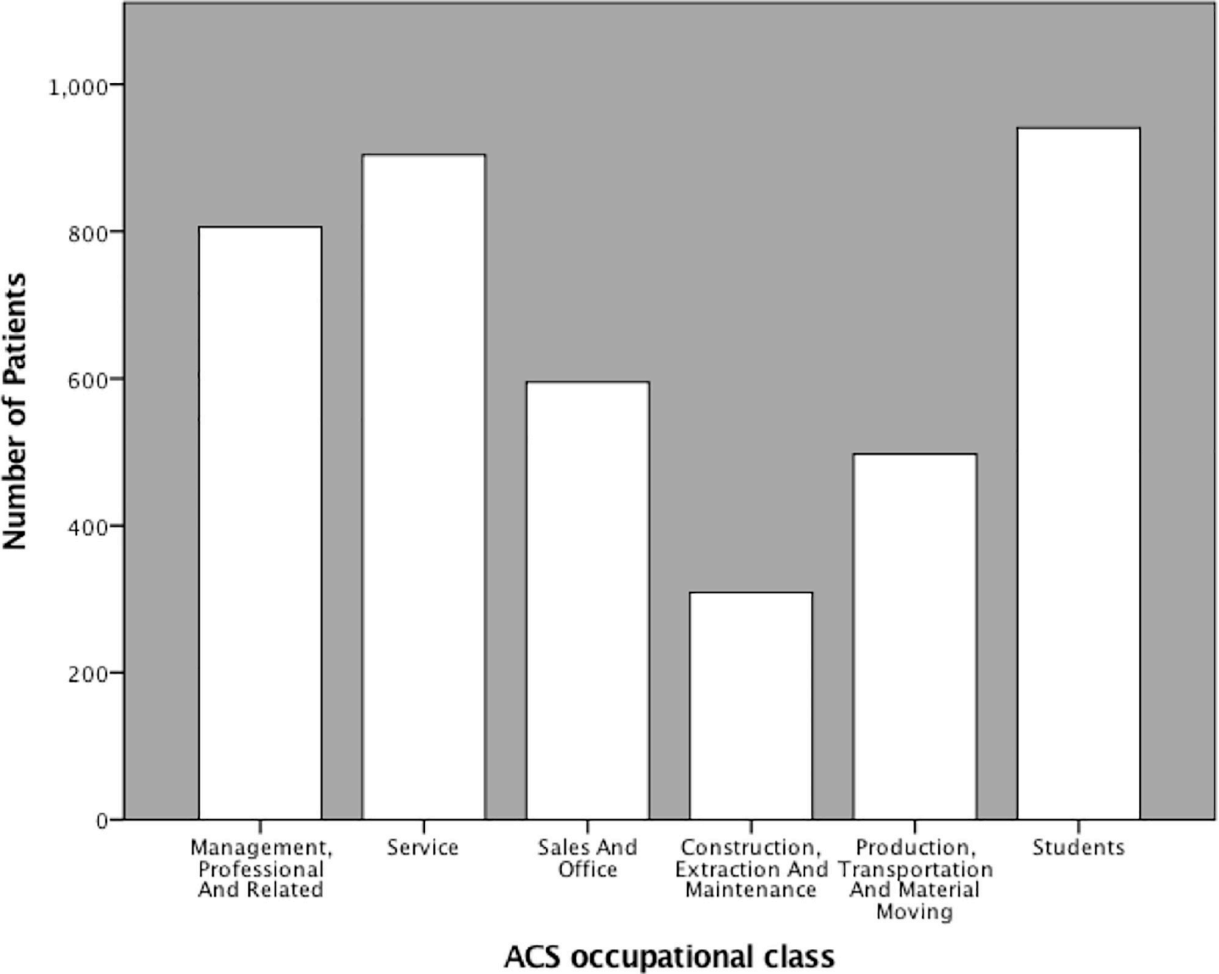
Distribution of occupational class.

Fig 1 depicts the distribution of patients in each ACS occupational class, and

**Fig 2:**
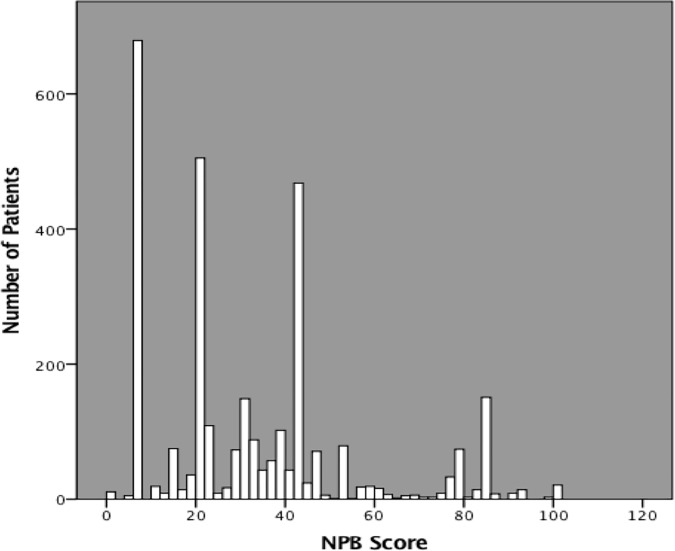
NPB score distribution.

Fig 2 depicts the distribution of NPB scores. The majority of patients (84%) fell below range midpoint of 50.

Table 2 reports the association between occupational environment and presenting diagnosis, which represents the direct effect of occupation on illness and injury events. Workers in Management, Professional and Related occupations (which includes farmers) were most commonly afflicted by noncommunicable disease, followed by trauma. The Service profession exhibited a similar pattern. Students, those in the Construction, Extraction and Maintenance, and those in Production, Transportation and Material Moving occupations mostly presented with injuries. Mental health was most prevalent amongst those in the Service industry, followed by students. We found significant associations for all sectors except for the “Sales And Office” class.

**Table 2:** Occupational environment by diagnosis.

	**Diagnoses**					
2010–2012 Occupational Environment n (row %)	Trauma n = 2273	Infectious Disease n = 754	Mental Health n = 506	Neoplasm n = 63	Noncommunicable n = 1542	**P-value**
**Management, Professional And Related** **N = 806**	292(36.2)	136(16.8)	37(4.5)	21(2.6)	312(38.7)	**<0.001**
**Service** **N = 904**	247(27.3)	122(13.4)	130(14.3)	12(1.3)	373(41.2)	**<0.001**
**Sales And Office** **N = 595**	271(45.5)	81(13.6)	65(10.9)	6(1)	169(28.4)	**0.662**
**Construction, Extraction And Maintenance** **N = 309**	179(57.9)	41(13.2)	23(7.4)	3(0.9)	57(18.4)	**<0.001**
**Production, Transportation And Material Moving** **N = 497**	290(58.3)	53(10.6)	26(5.2)	2(0.4)	118(23.7)	**<0.001**
**Students** **N = 942**	494(52.4)	158(16.7)	127(13.4)	2(0.2)	153(16.2)	**<0.001**

After examining the associations between age, sex, diagnosis and work environment (as defined by the highest hierarchical level of occupational category by ACS taxonomy) on outcome, we then performed a multivariate regression analysis adjusting for these factors.

**Fig 3:**
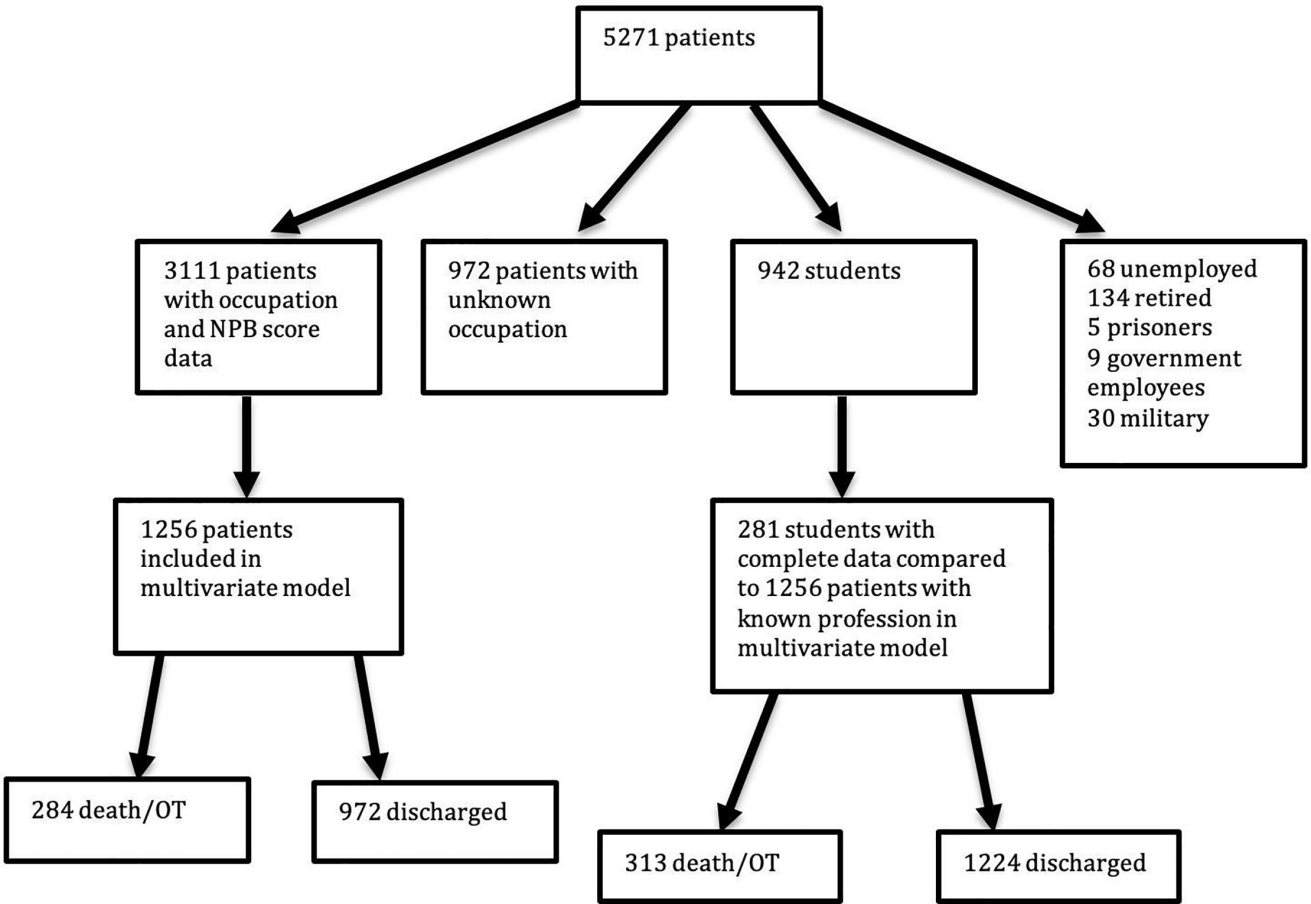
Data flow diagram.

Fig 3 is our data flow diagram for the following regression analyses.

Table 3 displays the results of this model, examining the independent effect of NPB score on the likelihood of death/OT outcome. Our large sample size allowed for us to observe statistically significant differences for each aforementioned variable. Relative to the 1^st^ quartile, the odds ratio for death/OT in the 4^th^ quartile was 2.9 (p = 0.004), the 3^rd^ quartile was 1.8 (p = 0.094), and the 2^nd^ quartile was 3.1 (p<0.001).

**Table 3:** Adjusted effect of SES quartile on poor outcome.

	OR	p-value	CI 95%
**NPB Quartile** **(1^st^ is reference group)**			
**1^st^ quartile (>43)**	reference	<0.001	
**2^nd^ quartile (29–42)**	3.1	<0.001	1.9–5.1
**3^rd^ quartile (16–28)**	1.8	0.094	0.9–3.5
**4^th^ quartile (<15)**	2.9	0.004	1.4–5.9
**Male sex**	1.836	0.006	1.187–2.837
**Age**	1.039	<0.001	1.027–1.050
**Diagnosis**			
**Trauma**	reference	<0.001	
**Infectious Disease**	19.499	<0.001	12.072–31.493
**Noncommunicable Disease**	13.862	<0.001	9.241–20.794
**ACS OCC Class**			
**Management, Professional And Related**	reference	0.025	
**Service**	0.379	0.004	0.195–0.736
**Sales And Office**	0.740	0.391	0.371–1.474
**Construction, Extraction And Maintenance**	0.649	0.237	0.316–1.329
**Production, Transportation And Material Moving**	0.461	0.008	0.261–0.814

We had a sizeable student population in this study (n = 942). Students do not have a corresponding NPB score or occupational class, therefore we performed a separate model (Table 4) to examine the independent effect of student status on the likelihood of death/OT outcome. We had sufficient sample size (281) to adjust for sex, age, and diagnosis. Being a student appears to have a protective effect, with an odds ratio of 0.55 (p = 0.022).

**Table 4:** Adjusted effect of being a student on poor outcome.

	**OR**	**P-value**	**CI 95%**
**Student (vs other occupations)**	0.550	0.022	0.330–0.917
**Male sex (vs female)**	1.980	<0.001	1.422–2.756
**Age (years)**	1.036	<0.001	1.025–1.048
**Trauma**	0.026	<0.001	0.006–0.115
**Infectious Disease**	21.595	<0.001	13.994–33.324
**Noncommunicable disease**	13.589	<0.001	9.352–19.746

## Discussion

As we move into the Sustainable Development Goal era, increasing emphasis is being placed not just on increasing access to care in LMICs, but also to improving the quality of the care being delivered. Indeed, equity across SES classes is a critical component of quality. The 2018 Lancet Global Health Commission on High Quality Health Systems states that “A focus on equity means that high-quality health care needs to be available and affordable for all people, regardless of underlying social disadvantages. Measures of quality need to be disaggregated by stratifiers of social power–such as wealth, gender, or ethnicity”[9] This paper takes the first step in disaggregating acute disease outcomes by such a stratifier in the Boeny region. It is vital that we expand the literature base describing such disparities in order to ensure that they are considered in future Malagasy emergency care systems development.

Table 1 outlines the demographic characteristics and emergency department diagnoses of our patients. Median age was 31 years. We saw an overall male predominance, and more males than females died or went to the operating theater. These findings are both in keeping with other regional reports from Uganda and Tanzania.[10,11] A disproportionately high injury burden in LMICs has been well-established by the Global Burden of Disease project, and our findings support that–with injury being the most common overall patient presentation.[12] The rate of discharge was highest amongst injured patients relative to other disease categories.

The distribution of patients in each occupational class is shown in Fig 1. The largest employed group with a known occupation was the Service industry, mostly comprised of housekeepers (ACS Occ Code 4230) and protective service occupations, which included security guards (ACS Occ Codes 3700–3955). This class was followed by those in Management, Professional and Related owing to the large number of independent farmers (ACS Occ Code 42) and teachers (ACS Occ Codes 2200–2340). To our knowledge there are no reports on the occupational class distribution of an emergency care population from a low, middle, or high-income country–most reports are either disease-specific such as asthmatics and stroke patients, or are tied to utilization rather than outcome.[4,5,13,14] In this respect, our study provides a novel perspective on these populations.

Fig 2 shows the NPB score distribution. The majority of patients (84%) fell into the lower half (<50) of the NPB score range. This distribution was similar to reports using other non-standardized methods of quantifying socioeconomic status such as level of education amongst hypertensive patients in Nigeria, Kenya, Tanzania and Namibia, and reported occupation in patients from the Seychelles.[3,15]

We found statistically significant correlations between ED diagnosis and all occupational environments except for Sales and Office. This likely reflects the absence of specific occupational hazards associated with that environment. This finding highlights the importance of controlling for this measure when assessing the impact of SES on patient outcome.

We had sufficient statistical power to examine the effect of socioeconomic status on outcome while adjusting for age, sex, diagnosis, and occupational class. Each of these factors was found to impact outcome in both univariate (p-value threshold <0.20) and multivariate (p-value threshold <0.05) modeling. We found statistically significant odds ratios ranging from 2.9 in the 4^th^ quartile to 3.1 in the highest quartile. The 3^rd^ quartile approached, but did not achieve significance in this model, with a odds ratio of 1.8 but confidence interval ranging from 0.9–3.5. While there are a range of studies examining the impact of SES on health in other settings, we found no studies examining the effect of SES on all-cause ED mortality–though there are condition-specific ED reports using a range of SES markers. Insurance status has been postulated as a surrogate marker, with a higher rate of trauma mortality (4.3%) in self-pay patients in Arizona, USA compared to private insurance (1.9%), p<0.0001.[16] Another study from Michigan, USA found no association between SES indicated by census-estimated median household income and increased mortality amongst patients with cardiac arrest (OR = 1.51,95% CI 0.8, 2.8).[17] One possible reason for our findings to show a greater impact than found in HICs is the Gini coefficient–the most commonly used measure of a country’s skew in income distribution–is higher in LMICs than in their HIC counterparts, indicating a wider income inequality gap and subsequent magnification of the relative deprivation of the lowest income tiers from the highest, which has an established effect on health.[8,18]

Students lack a defined ACS Occupational Code and therefore have no corresponding NPB score. Our student population was large enough (n = 942) to warrant independent investigation. Our model found that student status was protective even once adjusted for age. There are no comparable reports on the impact of student status on emergency department outcome in low, middle, or high-income countries. This finding is most likely due to their younger average age and presumed lower rate of comorbidities, but could also be due to their relative proximity to healthcare facilities (thereby improving access), insurance status, or other factors. Further investigation via dedicated study of the societal determinants of health of Malagasy students is needed to explore all possibilities.

Emergency care systems often serve as one of the first points of access to healthcare in low socioeconomic status populations. At their best, high-quality emergency care systems improve health equity by treating all comers, irrespective of social status or ability to pay. Such systems will be vital players in the Sustainable Development Goal era push towards universal health coverage (UHC) and will be integral in fulfilling the World Health Organization’s goal “to ensure that UHC reaches the poorest, the most marginalized, women, children, and people with disabilities”.[18, 19] Our findings identify an important emergency care disparity that we hope will serve as a call to action to policymakers and facility directors.

### Limitations

We coded occupation using American Community Survey occupational classification system codes. Using codes derived from an American population is a limitation, however there is no IPUMS occupational coding system for Madagascar. There is a coding system for its nearest neighbor Mozambique, which lists 119 distinct professional codes. By contrast, the ACS system lists 474 distinct codes. Furthermore, the ACS codes contain nearly all of the Mozambique system’s options, and where the two systems differed we found easily translatable equivalents (e.g. “324 Traditional medicine practitioners and faith healers” which we classified as “3540 Other Healthcare Practitioners and Technical Occupations”). Additionally, the NPB scoring system was derived from this scale; therefore in order to perform this analysis the ACS codes are required. In summary, using the ACS system was necessary to use the NPB scale, and gave us the added benefit of allowing more granular data on occupation itself. Finally, Fig 3. Data Flow Diagram shows that there were 972 out of 5271 patients that did not have a listed occupation (18%). The completeness of the remainder of their charts was similar to patients with a recorded occupation, therefore we feel that this was a nondifferential information bias.

## Conclusions

We found a significant association between lower socioeconomic status measured on an international standardized scale and poor emergency department outcome after controlling for sex, age, occupational environment and diagnosis. Our findings help identify a population vulnerable to poor outcomes from emergency conditions. We hope these findings can help identify patients at risk and facilitate the protection of vulnerable Malagasy populations, identified by the government as a key priority in Madagascar Action Plan.

## Supporting information

S1 File Anonymized dataset. (XLS)

S1 Table Top causes of mortality in Madagascar. (TIFF)
